# Associations of nutrient intake, dietary behaviours, and patterns with metabolic profiles and obesity measures: findings from a cross-sectional study in a southern Chinese population

**DOI:** 10.3389/fnut.2025.1586106

**Published:** 2025-08-01

**Authors:** Yifan Chen, Guanlin Chen, Yinglin Liang, Haiyan Huang, Yefeng Cai, Xiaojia Ni

**Affiliations:** ^1^The Second Clinical School of Guangzhou University of Chinese Medicine, Guangzhou, China; ^2^State Key Laboratory of Traditional Chinese Medicine Syndrome/State Key Laboratory of Dampnesss Syndrome of Chinese Medicine, The Second Affiliated Hospital of Guangzhou University of Chinese Medicine (Guangdong Provincial Hospital of Chinese Medicine), Guangdong Provincial Academy of Chinese Medical Sciences, The Second Clinical School of Guangzhou University of Chinese Medicine, Guangzhou, China

**Keywords:** dietary pattern, nutrient, metabolism, obesity, cross-sectional study

## Abstract

**Background:**

Metabolic diseases and obesity are highly prevalent, and diet plays a key role in their prevention and management. This study aimed to characterise the nutrient intake, dietary behaviours, and patterns of the South Chinese population and examine their associations with metabolic profiles and obesity measures.

**Methods:**

Data for this study were obtained from a cross-sectional study involving participants residing in Guangdong Province, China. Demographic information, disease history, nutrient intake, and dietary behaviours were collected via face-to-face interviews using structured questionnaires. Metabolic profiles and obesity levels were assessed via clinical laboratory tests, physical examinations, and bioelectrical impedance analysis. Principal component factor analysis (PCFA) was used to identify dietary patterns, while descriptive statistics, correlation analysis, and binary logistic regression were employed to characterise diets and assess their associations with metabolic profiles and obesity measures.

**Results:**

A total of 330 participants were included in this study, with a mean age of 53.62 years. Males accounted for 50.9% of the participants. The majority of participants preferred rice as their staple food and regularly consumed fresh vegetables. Red meat was frequently eaten, while white meat was consumed often. Seafood, legumes, and Cantonese soup were consumed occasionally, whereas traditional Chinese ultra-processed foods such as dairy and pasta were rarely consumed. Three distinct dietary patterns were identified in the study. The modern Cantonese dietary pattern was characterised by the consumption of white meat, eggs, milk, aquatic products, fresh fruits, vegetables, and Cantonese slow-cooked soup. The traditional Cantonese dietary pattern was defined by a high intake of traditional Chinese ultra-processed foods. Meanwhile, the localised Western dietary pattern featured the consumption of pasta, breakfast foods, coffee, and Cantonese desserts. The modern Cantonese dietary pattern was associated with a lower likelihood of dyslipidaemia than the traditional Cantonese dietary pattern, while the localised Western dietary pattern was linked to a reduced likelihood of glucose metabolism disorders and visceral obesity. Notably, these associations remained significant even among participants without a prior diagnosis of diabetes.

**Conclusion:**

This study characterised the dietary patterns of the South Chinese population and found that modern Cantonese dietary patterns appeared to be associated with lower odds of dyslipidaemia, while localised Western dietary patterns were potentially linked to a reduced likelihood of glucose metabolism disorders or visceral obesity.

## 1 Introduction

Metabolic diseases, including diabetes, hyperlipidaemia, and obesity, substantially contribute to the global disease burden (1). Globally, approximately 529 million people live with diabetes (2), and hypercholesterolaemia is estimated to account for 88 million disability-adjusted life years (3). In addition, obesity affects 603.7 million adults worldwide (4). These metabolic diseases are also major risk factors for cardiovascular diseases (CVDs) (5), due to the intricate interplay among inflammation, oxidative stress, endothelial dysfunction, lipid metabolism, and immune responses (6). In 2019, metabolic factors were responsible for 13.7 million CVD-related deaths, representing 73.7% of all CVD-related deaths (7). Dietary intervention is widely recognised as a key strategy for preventing metabolic diseases (8, 9). Recently, there has been growing interest in the role of dietary patterns in health promotion and disease prevention. Dietary patterns reflect the overall combination and quantities of foods and beverages that individuals habitually consume over time (10). Due to the complex interactions and synergistic effects of specific food combinations, dietary patterns play a crucial role in the prevention and management of metabolic diseases (8). For instance, the Mediterranean dietary pattern, commonly followed in the Mediterranean region, has been widely implemented and demonstrated to benefit metabolic health (11). However, since cultural traditions and dietary habits considerably influence food choices (12), these well-established dietary patterns may not be universally applicable. Investigating dietary patterns that align with local eating habits could enhance long-term adherence and effectiveness among regional populations.

In China, the prevalence of metabolic disease has risen considerably in recent years (13–15). Epidemiological studies indicate that Guangdong Province, located in southern China, has a higher prevalence of metabolic diseases than other regions. For instance, the estimated prevalence of diabetes in Guangdong province ranges from 15.4% to 22.1% (16). In addition, Guangdong has the highest reported prevalence of high total cholesterol (TC) and low-density lipoprotein cholesterol (LDL-C), with 16.7% of the population having high TC and 19.7% having high LDL-C levels (17).

The Cantonese dietary pattern, rooted in the region’s abundant aquatic and agricultural resources, is shaped by the long-standing culinary tradition that emphasises freshness, diversity, and nutritional balance (18–20). This diet, which is characterised by a wide variety of fresh vegetables, lean meats, and seafood, provides essential nutrients while being low in processed foods (18). Previous research has demonstrated the benefits of the Cantonese dietary pattern, including reduced all-cause and CVD mortality (18). However, some adverse effects have also been identified, such as a potential association with nasopharyngeal carcinoma (21). Despite these findings, the relationship between Cantonese dietary patterns and metabolic diseases remains unclear. Therefore, it is crucial to further investigate variations in dietary habits among Cantonese populations and identify dietary patterns that promote metabolic health. In this study, we employed a cross-sectional study design to analyse data on potential associations between dietary habits and metabolic indicators among individuals aged ≥40 years in Guangdong Province to explore the impact of dietary patterns on metabolic diseases in this region, providing insights for dietary interventions aimed at preventing and managing metabolic diseases.

## 2 Materials and methods

### 2.1 Study design and populations

The data for this study were obtained from a cross-sectional survey purposively recruited individuals aged ≥40 years residing in Guangdong Province for >6 months (22). The study was approved by the Ethics Committee of Guangdong Provincial Hospital of Chinese Medicine (Institutional Approval No. BE2021-195), and all participants provided written informed consent. This study adhered to the Strengthening the Reporting of Observational Studies in Epidemiology (STROBE) checklist (23). A combination of questionnaire surveys, physical examinations, and laboratory tests was used to comprehensively evaluate the association between dietary patterns and metabolic indicators in the Guangdong population.

### 2.2 Dietary assessment

Dietary information was collected using a food frequency questionnaire (FFQ). The FFQ included 22 food items, which were grouped into seven categories based on nutrient profiles or cultural similarities. These categories included staple food (such as rice and noodles); meat, poultry, and eggs (such as red meat, white meat, aquatic products, and eggs); plant-based foods (such as fresh vegetables, fresh fruits, and legumes); beverages (such as milk, tea, and coffee); ultra-processed foods (such as smoked and dried meat products, pickles, roasted meat, spiced corned food, and fried food); Western food (such as pizza and pasta); and Cantonese speciality foods (such as Cantonese slow-cooked soup, herbal tea, and Cantonese desserts). Participants were asked to report their consumption frequency using the following options: “never,” “1–3 times/month,” “1–3 days/week,” “4–6 days/week,” and “every day.” Responses were recorded on a scale of 1 to 5, with 1 representing the lowest frequency and 5 the highest. The interviewer received training prior to the survey and the questionnaire was tested to ensure its reliability and feasibility.

To identify dietary patterns, the consumption frequencies of 22 food items were included in the principal component factor analysis (PCFA) with varimax rotation. The eigenvalues (>1), scree plot, and factor interpretability were used to determine the number of factors (dietary patterns) to retain. Rotated factor loadings with an absolute value ≥ |0.30| were considered to significantly contribute to the dietary pattern, and higher values of factor loadings represented a stronger association between food groups and dietary patterns. Rotated factor loadings with an absolute value < |0.30| were omitted.

### 2.3 Measurement of metabolic profiles

Fasting venous blood samples (after ≥10 h of fasting) were collected on-site by a team of nurses. Triglyceride (TG) and TC levels were measured using the glycerol phosphate oxidase – phenol aminophenazone enzyme assay, while LDL-C and high-density lipoprotein cholesterol (HDL-C) levels were determined via enzymatic colorimetric assays. Fasting plasma glucose (FPG) levels were measured using the hexokinase method, and glycated haemoglobin (HBA1c) levels were assessed using capillary electrophoresis or borate affinity high-performance liquid chromatography. Anthropometric measurements were obtained by nurses. Body height was measured to the nearest 0.5 cm, with participants standing barefoot. Body weight was measured to the nearest 0.1 kg while participants wore light clothing. Waist circumference (WC) and hip circumference were measured to the nearest 0.5 cm. Body mass index (BMI) was calculated as weight (kg) divided by the square of height (m^2^). The waist-to-height ratio (WHtR) was calculated as the ratio of WC to height, and the waist-to-hip ratio (WHR) was calculated as the ratio of WC to hip circumference. The visceral adiposity index (VAI), a sex-specific algorithm, was calculated using WC, BMI, TG concentration, and HDL-C concentration with the following formula (24):


(1) $$V\,A\,I\,\left(m\,a\,l\,e\right)=\left(\frac{W\,C}{39.68+\left(1.88\times B\,M\,I\right)}\right)\times \left(\frac{T\,G}{1.03}\right)\times \left(\frac{1.31}{H\,D\,L-C}\right)$$

(2) $$V\,A\,I\,\left(f\,e\,m\,a\,l\,e\right)=\left(\frac{W\,C}{36.58+\left(1.89\times B\,M\,I\right)}\right)\times \left(\frac{T\,G}{0.81}\right)\times \left(\frac{1.52}{H\,D\,L-C}\right)$$

Body composition was assessed using the InBody 770 body composition analyser (InBody Co., Ltd., South Korea). This device employs bioelectrical impedance analysis (BIA) to measure the percentage of body fat (PBF), visceral fat area (VFA), and skeletal muscle mass. Prior to the examination, patients were instructed to maintain a fasting state, remain at rest in the early morning, remove all metallic items, as well as gloves, socks and shoes, and ensure no metal implants or electronic devices (e.g., pacemakers) were present. A weak alternating current is introduced into the body, and the resulting impedance values are used to estimate body water volume (25, 26). Out of the 330 participants, 238 underwent body composition examinations using the InBody device.

### 2.4 Ascertainment of metabolic conditions

Dyslipidaemia was defined according to the 2016 Chinese guidelines for the management of dyslipidaemia in adults, with one or more of the following criteria: TG ≥ 2.26 mmol/L, TC ≥ 6.22 mmol/L, LDL-C ≥ 4.14 mmol/L, or HDL-C < 1.04 mmol/L (27, 28). Diabetes was defined as FPG ≥ 7 mmol/L or HBA1c ≥ 6.5%, based on the Standards of Care in Diabetes—2024 (29). Prediabetes was defined as FPG of 5.6–6.9 mmol/L or HBA1c of 5.7–6.4%, in accordance with the diagnostic criteria established by the American Diabetes Association (29). Obesity was defined as a BMI ≥ 30 kg/m^2^, following the World Health Organization’s definition (30). Abdominal obesity was defined as a WC ≥ 90 cm for men or ≥85 cm for women, based on previous studies (31). An abnormal WHR was defined as WHR ≥ 0.9 for men or ≥0.85 for women, and an abnormal WHtR was defined as WHtR ≥ 0.5 (32). PBF ≥ 25% in men or ≥35% in women was classified as obesity (33). Visceral obesity was defined as a VFA ≥ 80 cm^2^ (34), and an abnormal VAI was defined as VAI > 1 abnormal (24, 35).

### 2.5 Statistical analysis

Descriptive statistics were used to analyse the distribution of habitual dietary variables and the basic characteristics of the population, categorised by dietary patterns. For statistical tests of differences, the Chi-square test was applied to categorical variables, and one-way ANOVA was used for continuous variables. Spearman’s rank correlation analysis was employed to evaluate the relationship between dietary habits and metabolic indicators. Binary logistic regression was used to examine the association between each dietary pattern and metabolic profiles or obesity measures, adjusting for covariates such as sex, age, smoking status, alcohol consumption, exercise status, and previous diagnoses of hypertension, and diabetes. The results are presented as odds ratios (ORs) and 95% confidence intervals (CIs). A sensitivity analysis was conducted by excluding participants with a prior diagnosis of diabetes, as its management and treatment could potentially introduce reverse causality. All statistical analyses and visualisations were performed using IBM SPSS Statistics version 25 (SPSS Inc., Chicago, IL, USA) and R 4.2.2. A *p-*value of <0.05 was considered statistically significant.

## 3 Results

### 3.1 Study participants

The study included a total of 330 participants, with a mean age of 53.62 years, of whom 50.9% were male. All participants were currently residing in Guangzhou, and 128 (38.8%) were born in Guangdong. Every participant had lived in Guangdong for >6 months.

**FIGURE 1:**
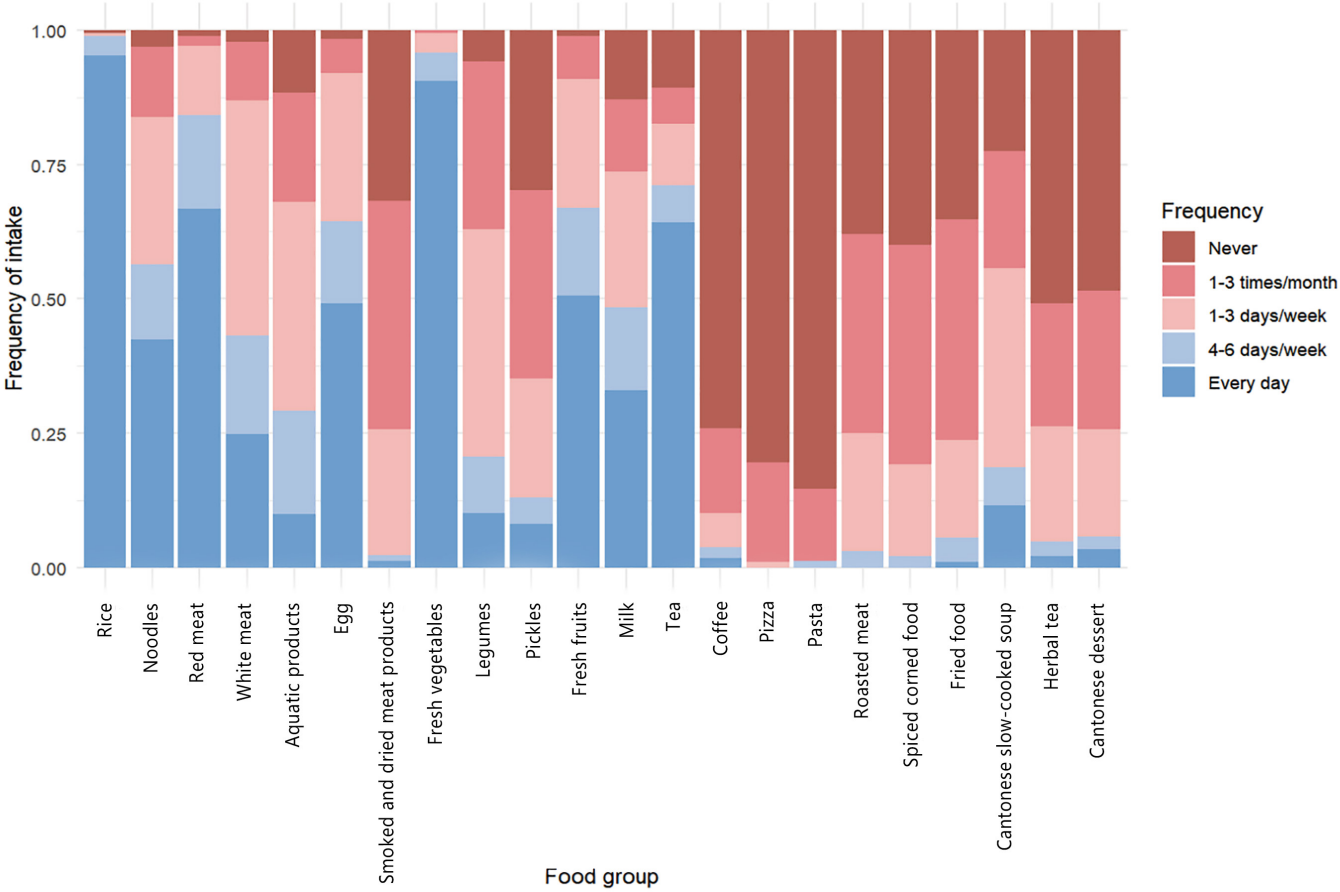
Overall dietary habits of participants. This figure shows the percentage distribution of food consumption frequency among participants. The colours of the bar graph represent the five frequency categories: “never,” “1–3 times per month,” “1–3 days per week,” “4–6 days per week,” and “every day.”

The dietary habits of the participants are shown in Figure 1. The majority (92.1%) consumed rice as their staple food daily; 56.4% ate red meat daily, and 45.8% consumed white meat daily. Legumes were eaten 1–3 times per week by 38.5% of participants, and 32.7% consumed aquatic products 1–3 times per month. Fresh vegetables were a daily staple for 86.7% of participants. Ultra-processed foods, such as pickles, smoked and dried meat products, and roasted meat, as well as Western foods like breakfast items and pasta, were rarely consumed. Similarly, foods with Cantonese characteristics, such as Cantonese slow-cooked soup, were consumed infrequently.

### 3.2 Dietary pattern

The foods included in the FFQ were analysed using PCFA. The Kaiser–Meyer–Olkin statistic for the model was 0.667, and Bartlett’s test of sphericity yielded a *p-*value < 0.001, indicating that the dietary frequency data were suitable for PCFA.

**FIGURE 2:**
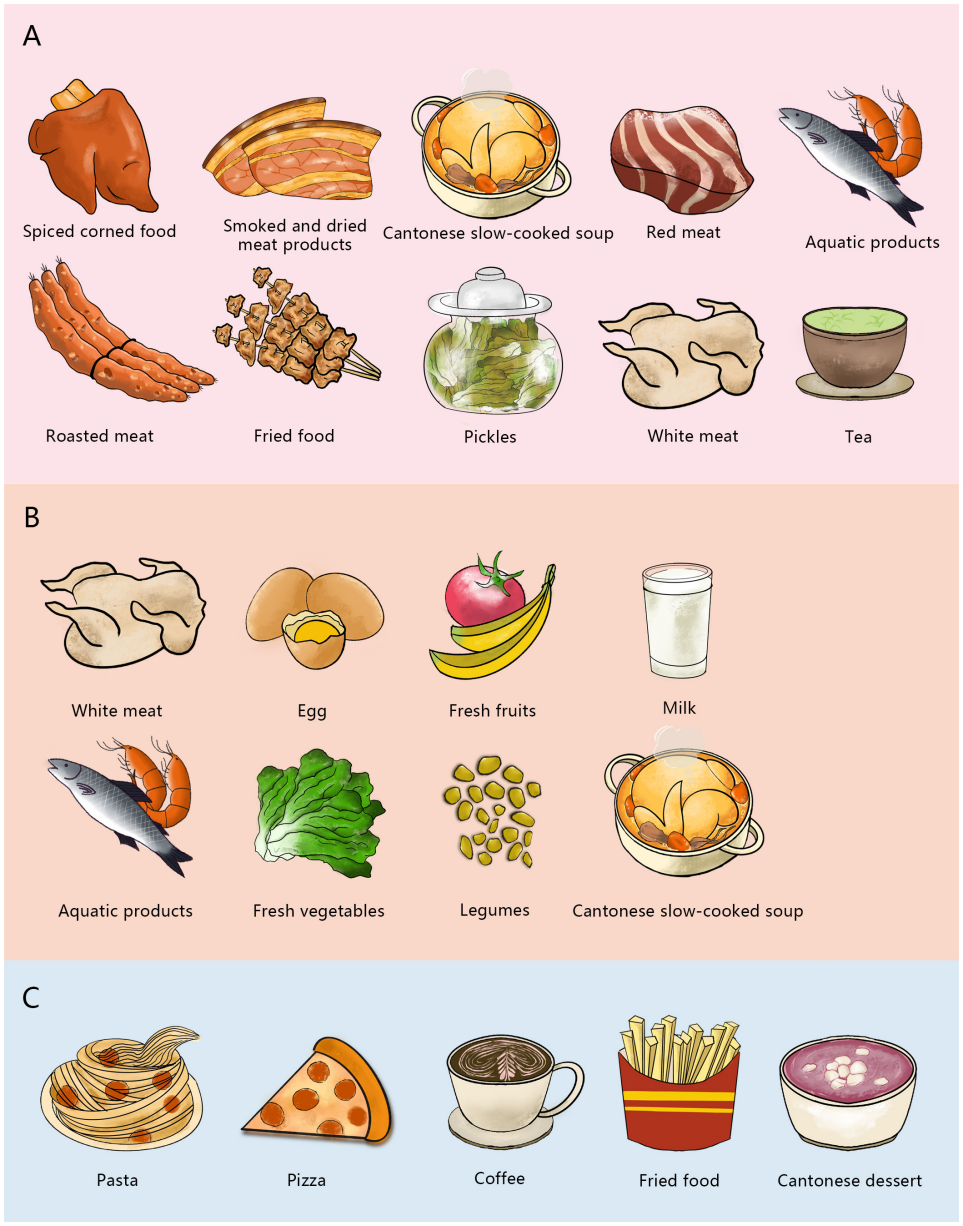
Dietary patterns. This figure presents the components of the three dietary patterns identified through factor analysis. **(A)** Traditional Cantonese dietary pattern. **(B)** Modern Cantonese dietary pattern. **(C)** Localised Western dietary pattern. This figure was drawn by Yifen Chen with Procreate.com.

Three major dietary patterns were identified through PCFA (Table 1 and Figure 2), and dietary patterns were named based on food groups with high factor loadings (rotated factor loadings with an absolute value ≥ |0.30|). The first, the traditional Cantonese dietary pattern, explained 13.9% of the variance and was characterised by the consumption of traditional Chinese ultra-processed foods, such as pickles, smoked and dried meat products, and roasted meat. The second, the localised Western dietary pattern, explained 6.8% of the variance and was characterised by the consumption of Western-style foods, such as pizza, pasta, and coffee, though less frequently than in Western countries, along with Cantonese desserts. The third, the modern Cantonese dietary pattern, explained 9.1% of the variance and retained traditional Cantonese dietary habits, such as consuming aquatic products, fresh fruits, vegetables, and slow-cooked soup, while incorporating modern healthy dietary practices, such as eating white meat, eggs, milk, and legumes. The basic characteristics of participants across the three dietary pattern groups are presented in Table 2. Among the groups, 116 participants followed the traditional Cantonese dietary pattern, 89 followed the localised Western dietary pattern, and 125 followed the modern Cantonese dietary pattern. Participants adhering to the modern Cantonese dietary pattern were more likely to be female and older, with lower rates of smoking and alcohol consumption. They were also less likely to have a history of dyslipidaemia or stroke.

**TABLE 1:** Dietary patterns and their factor loading determined using factor analysis.^a^

Food items	Traditional Cantonese dietary pattern	Modern Cantonese dietary pattern	Localised Western dietary pattern
Spiced corned food	0.645		
Roasted meat	0.632		
Red meat	0.491		
White meat	0.483	0.354	
Cantonese slow-cooked soup	0.430	0.350	
Smoked and dried meat products	0.418		
Pickles	0.414		
Tea	0.346		
Fresh fruits		0.646	
Milk		0.610	
Egg		0.539	
Aquatic products	0.372	0.416	
Fresh vegetables		0.386	
Legumes		0.328	
Pizza			0.778
Pasta			0.709
Cantonese desserts			0.503
Fried food	0.401		0.461
Coffee			0.314

*^a^*Factors were rotated via the varimax rotation; rotated factor loadings with an absolute value < |0.30| were omitted.

**TABLE 2:** Basic characteristics of the participants according to the three dietary patterns.

Variables	Traditional Cantonese dietary pattern	Modern Cantonese dietary pattern	Localised Western dietary pattern	*p*-Value^a^
**Sex, n (%)**				
Male	77 (66.4)	55 (44.0)	36 (40.4)	<0.001
Female	39 (33.6)	70 (56.0)	53 (59.6)	
Age, mean (SD)	53.93 (7.63)	54.59 (8.68)	51.87 (7.26)	0.042
**Smoking status, n (%)**				
Yes	53 (45.7)	33 (26.4)	16 (18.0)	<0.001
No	63 (54.3)	92 (73.6)	73 (82.0)	
**Alcohol intake, n (%)**				
Yes	53 (45.7)	28 (22.4)	22 (24.7)	<0.001
No	63 (54.3)	97 (77.6)	67 (75.3)	
**Lack of exercise, n (%)**				
Yes	9 (7.8)	14 (11.2)	9 (10.1)	0.658
No	107 (92.2)	111 (88.8)	80 (89.9)	
**Lipid profile, mean (SD)**				
TG	1.97 (2.32)	1.56 (1.47)	1.70 (1.04)	0.185
TC	5.10 (1.03)	5.04 (0.98)	5.00 (0.89)	0.730
HDL-C	1.24 (0.32)	1.37 (0.30)	1.28 (0.32)	0.007
LDL-C	3.24 (0.90)	3.20 (0.92)	3.15 (0.73)	0.736
FPG, mean (SD)	5.95 (1.92)	5.81 (2.38)	5.21 (1.33)	0.023
HBA1c, mean (SD)	5.99 (1.23)	5.79 (0.84)	5.93 (1.24)	0.341
BMI, mean (SD)	25.36 (3.01)	24.48 (3.14)	25.14 (4.20)	0.118
WC, mean (SD)	89.05 (8.35)	86.07 (8.17)	87.49 (9.95)	0.032
WHtR, mean (SD)	0.54 (0.05)	0.54 (0.05)	0.54 (0.06)	0.345
WHR, mean (SD)	0.90 (0.06)	0.89 (0.06)	0.88 (0.06)	0.034
**Dyslipidaemia, n (%)**				
Yes	66 (56.9)	47 (37.6)	46 (51.7)	0.008
No	50 (43.1)	78 (62.4)	43 (48.3)	
**Diabetes, n (%)**				
Yes	14 (12.1)	15 (12.0)	7 (7.9)	0.559
No	102 (87.9)	110 (88.0)	82 (92.1)	
**Hypertension, n (%)**				
Yes	44 (37.9)	42 (33.6)	32 (36.0)	0.781
No	72 (62.1)	83 (66.4)	57 (64.0)	
**Stroke, n (%)**				
Yes	0	4 (3.2)	0	0.036
No	116 (100.0)	121 (96.8)	89 (100.0)	

*^a^*ANOVA one-way test for continuous variables and Chi-square test for categorical variables. *p* < 0.05 was considered to indicate statistical significance. TC, total cholesterol; TG, triglycerides; LDL-C, low-density lipoprotein cholesterol; HDL-C, high-density lipoprotein cholesterol; FPG, fasting plasma glucose; HbA1c, glycated haemoglobin; BMI, body mass index; WC, waist circumference; VAI, visceral adiposity index; PBF, percentage of body fat; VFA, visceral fat area.

### 3.3 Associations between food consumption and metabolic profiles/obesity measures

**FIGURE 3:**
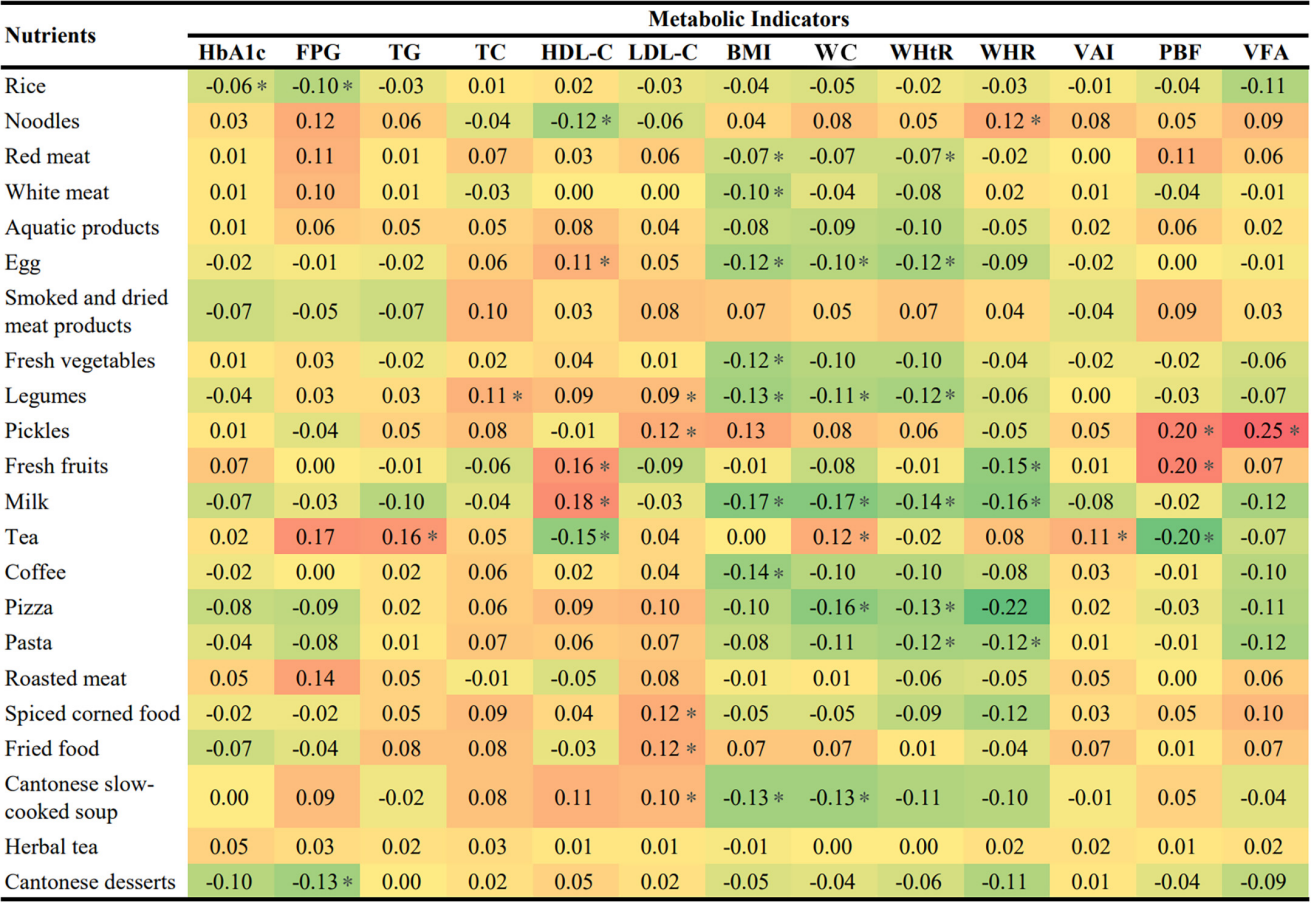
Correlation matrix for dietary habits and metabolic profiles. This figure displays the correlation strength between dietary habits and metabolic profiles. The colour of each square at the intersection of two variables represents the correlation strength, with bright red indicating a strong positive correlation and bright green indicating a strong negative correlation. *p < 0.05. TC, total cholesterol; TG, triglyceride; LDL-C, low-density lipoprotein cholesterol; HDL-C, high-density lipoprotein cholesterol; FPG, fasting plasma glucose; HbA1c, glycated haemoglobin; BMI, body mass index; WC, waist circumference; VAI, visceral adiposity index; PBF, percentage of body fat; VFA, visceral fat area.

Spearman rank correlations between the frequency of food consumption and metabolic profiles are shown in Figure 3. Notably, certain dietary components were correlated with lipid profiles, glucose levels, and obesity measures. Higher coffee intake was associated with a lower BMI, while greater pasta consumption was associated with higher WHtR and WHR. Consumption of fresh fruits and vegetables was correlated with reduced BMI and WHR. In addition, intake of eggs, milk, and white meat was correlated with lower obesity measures, while positive relationships were observed between HDL-C levels and the consumption of eggs and milk. Cantonese slow-cooked soups showed a weak association with higher LDL-C levels. Ultra-processed foods such as pickles and fried foods were also weakly associated with LDL-C. After excluding participants with diabetes, no significant correlations were found between the frequency of rice and Cantonese dessert consumption and metabolic profiles or obesity measures.

### 3.4 Associations between dietary pattern and metabolic profiles/obesity measures

To further explore the associations between dietary patterns, metabolic profiles, and obesity measures, binary logistic regression analyses were performed, adjusting for age, sex, smoking, alcohol consumption, exercise, and hypertension.

The results are presented in Table 3. Regarding dyslipidaemia, the modern Cantonese dietary pattern was associated with lower odds of low HDL-C levels in the fully adjusted model (OR = 0.453, 95% CI: 0.222–0.922) than the traditional Cantonese dietary pattern. For FPG, individuals adhering to the localised Western dietary pattern had a significantly lower likelihood of high FPG levels than those following the traditional ultra-processed food dietary pattern (OR = 0.197, 95% CI: 0.093–0.419). In terms of obesity, localised Western dietary patterns were associated with significantly reduced probabilities of excessive PBF (OR = 0.365, 95% CI: 0.173–0.767) and VFA (OR = 0.408, 95% CI: 0.192–0.869).

**TABLE 3:** Odds ratios and 95% confidence intervals of metabolic indicators according to dietary pattern.

	All population				Population without prior diabetes			
Metabolic profiles and obesity measures	Model	Traditional Cantonese dietary pattern	Localised Western dietary pattern	Modern Cantonese dietary pattern	Model	Traditional Cantonese dietary pattern	Localised Western dietary pattern	Modern Cantonese dietary pattern
		OR (95% CI)	OR (95% CI)	OR (95% CI)		OR (95% CI)	OR (95% CI)	OR (95% CI)
Hypercholesterolemia	Crude model	Ref.	0.617 (0.252–1.515)	0.852 (0.401–1.813)	Crude model	Ref.	0.740 (0.291–1.882)	0.918 (0.404–2.085)
	Model 1^a^		0.615 (0.245–1.544)	0.868 (0.402–1.876)	Model 1^a^		0.742 (0.286–1.925)	0.924 (0.402–2.126)
	Model 2^b^		0.619 (0.247–1.553)	0.858 (0.397–1.857)	Model 2^b^		0.746 (0.288–1.933)	0.921 (0.400–2.118)
	Model 3^c^		0.688 (0.269–1.760)	0.933 (0.423–2.058)	Model 3^c^		0.817 (0.309–2.159)	0.998 (0.426–2.340)
Hypertriglyceridemia	Crude model	Ref.	1.228 (0.614–2.456)	0.437 (0.200–0.951)*	Crude model	Ref.	1.072 (0.520–2.212)	0.279 (0.112–0.691)*
	Model 1^a^		1.336 (0.647–2.756)	0.493 (0.223–1.088)	Model 1^a^		1.153 (0.544–2.444)	0.306 (0.122–0.765)*
	Model 2^b^		1.328 (0.630–2.800)	0.514 (0.230–1.151)	Model 2^b^		1.147 (0.530–2.482)	0.309 (0.122–0.783)*
	Model 3^c^		1.487 (0.689–3.210)	0.554 (0.243–1.260)	Model 3^c^		1.252 (0.565–2.773)	0.330 (0.128–0.847)*
High LDL-C	Crude model	Ref.	0.703 (0.295–1.675)	0.852 (0.401–1.813)	Crude model	Ref.	1.020 (0.401–2.593)	1.109 (0.473–2.600)
	Model 1^a^		0.780 (0.319–1.908)	0.860 (0.397–1.865)	Model 1^a^		1.154 (0.443–3.005)	1.167 (0.490–2.777)
	Model 2^b^		0.786 (0.321–1.922)	0.837 (0.385–1.820)	Model 2^b^		1.168 (0.448–3.041)	1.151 (0.483–2.744)
	Model 3^c^		0.874 (0.350–2.178)	0.870 (0.392–1.931)	Model 3^c^		1.320 (0.495–3.520)	1.251 (0.512–3.057)
Low HDL-C	Crude model	Ref.	0.915 (0.489–1.710)	0.413 (0.215–0.794)*	Crude model	Ref.	0.838 (0.426–1.647)	0.372 (0.180–0.770)*
	Model 1^a^		1.216 (0.616–2.397)	0.530 (0.268–1.046)	Model 1^a^		1.022 (0.493–2.119)	0.449 (0.211–0.957)*
	Model 2^b^		1.221 (0.616–2.419)	0.542 (0.273–1.074)	Model 2^b^		1.031 (0.494–2.154)	0.455 (0.212–0.976)*
	Model 3^c^		1.122 (0.550–2.292)	0.453 (0.222–0.922)*	Model 3^c^		0.889 (0.409–1.933)	0.362 (0.163–0.805)*
FPG ≥ 5.6 mmol/L	Crude model	Ref.	0.186 (0.090–0.386)*	0.668 (0.396–1.126)	Crude model	Ref.	0.164 (0.068–0.393)*	0.600 (0.333–1.081)
	Model 1^a^		0.195 (0.093–0.410)*	0.683 (0.400–1.165)	Model 1^a^		0.171 (0.071–0.414)*	0.622 (0.342–1.132)
	Model 2^b^		0.192 (0.091–0.405)*	0.692 (0.405–1.183)	Model 2^b^		0.166 (0.068–0.405)*	0.627 (0.343–1.143)
	Model 3^c^		0.197 (0.093–0.419)*	0.738 (0.428–1.274)	Model 3^c^		0.174 (0.071–0.429)*	0.701 (0.377–1.304)
Fasting HbA1c ≥ 5.7%	Crude model	Ref.	0.789 (0.452–1.378)	1.202 (0.724–1.993)	Crude model	Ref.	0.844 (0.467–1.525)	1.271 (0.740–2.185)
	Model 1^a^		0.805 (0.452–1.435)	1.132 (0.673–1.906)	Model 1^a^		0.870 (0.472–1.602)	1.216 (0.698–2.118)
	Model 2^b^		0.810 (0.454–1.445)	1.119 (0.664–1.887)	Model 2^b^		0.875 (0.475–1.612)	1.210 (0.694–2.109)
	Model 3^c^		0.814 (0.451–1.469)	1.113 (0.653–1.898)	Model 3^c^		0.885 (0.473–1.655)	1.191 (0.673–2.106)
BMI	Crude model	Ref.	1.342 (0.533–3.379)	0.534 (0.488–1.520)	Crude model	Ref.	0.995 (0.374–2.647)	0.438 (0.144–1.329)
	Model 1^a^		1.172 (0.449–3.060)	0.526 (0.181–1.529)	Model 1^a^		0.877 (0.319–2.409)	0.430 (0.139–1.327)
	Model 2^b^		1.111 (0.413–2.986)	0.558 (0.189–1.650)	Model 2^b^		0.836 (0.296–2.360)	0.444 (0.142–1.395)
	Model 3^c^		1.276 (0.461–3.534)	0.637 (0.210–1.931)	Model 3^c^		0.967 (0.332–2.813)	0.504 (0.157–1.620)
WC	Crude model	Ref.	0.831 (0.478–1.446)	0.726 (0.437–1.206)	Crude model	Ref.	0.752 (0.420–1.347)	0.682 (0.397–1.173)
	Model 1^a^		0.902 (0.509–1.599)	0.802 (0.477–1.347)	Model 1^a^		0.822 (0.451–1.499)	0.750 (0.432–1.302)
	Model 2^b^		0.872 (0.486–1.566)	0.835 (0.491–1.419)	Model 2^b^		0.782 (0.422–1.449)	0.763 (0.433–1.345)
	Model 3^c^		0.870 (0.481–1.573)	0.836 (0.488–1.431)	Model 3^c^		0.788 (0.422–1.473)	0.776 (0.436–1.381)
WHR	Crude model	Ref.	0.903 (0.496–1.646)	0.737 (0.429–1.267)	Crude model	Ref.	0.804 (0.430–1.500)	0.701 (0.395–1.246)
	Model 1^a^		1.124 (0.600–2.106)	0.774 (0.440–1.361)	Model 1^a^		0.978 (0.511–1.872)	0.733 (0.404–1.330)
	Model 2^b^		1.097 (0.583–2.063)	0.794 (0.450–1.403)	Model 2^b^		0.948 (0.493–1.825)	0.745 (0.409–1.360)
	Model 3^c^		1.118 (0.591–2.116)	0.805 (0.453–1.429)	Model 3^c^		0.966 (0.498–1.873)	0.756 (0.412–1.389)
WHtR	Crude model	Ref.	0.487 (0.238–0.996)*	0.554 (0.282–1.088)	Crude model	Ref.	0.434 (0.206–0.915)*	0.489 (0.240–0.996)*
	Model 1^a^		0.568 (0.271–1.188)	0.561 (0.280–1.123)	Model 1^a^		0.503 (0.234–1.081)	0.505 (0.244–1.044)
	Model 2^b^		0.547 (0.260–1.151)	0.577 (0.287–1.160)	Model 2^b^		0.480 (0.222–1.038)	0.512 (0.246–1.065)
	Model 3^c^		0.527 (0.248–1.120)	0.574 (0.283–1.162)	Model 3^c^		0.469 (0.215–1.026)	0.514 (0.245–1.077)
PBF	Crude model	Ref.	0.344 (0.173–0.682)*	0.491 (0.269–0.899)*	Crude model	Ref.	0.359 (0.176–0.729)*	0.517 (0.274–0.976)*
	Model 1^a^		0.394 (0.194–0.797)*	0.556 (0.299–1.034)	Model 1^a^		0.410 (0.198–0.849)*	0.574 (0.300–1.097)
	Model 2^b^		0.405 (0.199–0.823)*	0.572 (0.306–1.068)	Model 2^b^		0.420 (0.202–0.873)*	0.588 (0.306–1.131)
	Model 3^c^		0.365 (0.173–0.767)*	0.564 (0.293–1.085)	Model 3^c^		0.399 (0.186–0.857)*	0.594 (0.298–1.181)
VFA	Crude model	Ref.	0.606 (0.317–1.160)	0.809 (0.446–1.468)	Crude model	Ref.	0.536 (0.273–1.054)	0.810 (0.433–1.516)
	Model 1^a^		0.450 (0.223–0.906)*	0.618 (0.328–1.167)	Model 1^a^		0.390 (0.187–0.812)*	0.624 (0.320–1.216)
	Model 2^b^		0.465 (0.226–0.955)*	0.647 (0.339–1.234)	Model 2^b^		0.395 (0.186–0.842)*	0.648 (0.328–1.279)
	Model 3^c^		0.408 (0.192–0.869)*	0.626 (0.316–1.240)	Model 3^c^		0.359 (0.162–0.793)*	0.651 (0.315–1.346)
VAI	Crude model	Ref.	1.105 (0.552–2.211)	0.698 (0.384–1.269)	Crude model	Ref.	1.176 (0.582–2.377)	0.718 (0.389–1.325)
	Model 1^a^		1.209 (0.593–2.466)	0.711 (0.386–1.310)	Model 1^a^		1.291 (0.628–2.652)	0.732 (0.392–1.367)
	Model 2^b^		1.182 (0.577–2.419)	0.731 (0.395–1.354)	Model 2^b^		1.257 (0.609–2.596)	0.743 (0.396–1.396)
	Model 3^c^		1.269 (0.614–2.624)	0.750 (0.400–1.405)	Model 3^c^		1.346 (0.644–2.813)	0.779 (0.410–1.481)

TC, total cholesterol; TG, triglycerides; LDL-C, low-density lipoprotein cholesterol; HDL-C, high-density lipoprotein cholesterol; FPG, fasting plasma glucose; HbA1c, glycated haemoglobin; BMI, body mass index; WC, waist circumference; VAI, visceral adiposity index; PBF, percentage of body fat; VFA, visceral fat area.

*p < 0.05.

*^a^*Adjusted for age and sex.

*^b^*Adjusted for age, sex, and hypertension.

*^c^*Adjusted for age, sex, smoking status, alcohol consumption, exercise, and hypertension.

To address potential reverse causality—where individuals might change their dietary habits due to a diagnosis of metabolic disease—logistic regression analyses were repeated after excluding participants with a previous diagnosis of diabetes (Table 3). These analyses revealed that adherence to the modern Cantonese dietary pattern was associated with significantly lower odds of high TG levels (OR = 0.330, 95% CI: 0.128–0.847) and low HDL-C levels (OR = 0.362, 95% CI: 0.163–0.805), while adherence to the localised Western dietary pattern was associated with significantly lower odds of high FPG levels (OR = 0.174, 95% CI: 0.071–0.429), excessive PBF (OR = 0.399, 95% CI: 0.186–0.857) and VFA (OR = 0.359, 95% CI: 0.162–0.793). No significant differences were observed in BMI, WC, WHR, or WHtR between dietary patterns.

## 4 Discussion

In this cross-sectional study of individuals aged >40 years in Guangdong, we identified three dietary patterns using PCFA: traditional Cantonese, modern Cantonese, and localised Western dietary patterns. The localised Western dietary pattern was associated with lower odds of elevated FPG levels and appeared to be more beneficial in reducing the risk of visceral obesity than the traditional Cantonese dietary pattern, while the modern Cantonese dietary pattern was associated with a decreased likelihood of dyslipidaemia. These findings provide insights into the potential health implications of different dietary patterns in Guangdong Province, highlighting opportunities for improved dietary interventions to prevent metabolic diseases.

The localised Western dietary pattern was particularly associated with better FPG control and reductions in both PBF and VFA. In contrast, the traditional Western diet is typically high in calories, with excessive intake of saturated fats, refined sugars (36), processed meats (37), and refined grains (38), while being low in unprocessed fruits, vegetables, whole grains, grass-fed animal products, and legumes (37). Previous studies have linked the traditional Western diet to an increased risk of type 2 diabetes (39, 40). The localised Western dietary pattern in our study featured a lower intake of processed meats and animal fats than the traditional Western diet and was instead characterised by the consumption of pasta and coffee. Previous studies have suggested that both pasta and coffee consumption are associated with a reduced risk of type 2 diabetes and overall obesity (41–43). Our study further supports these findings, indicating that the localised Western dietary pattern—incorporating both pasta and coffee—was associated with lower odds of high FPG, PBF, and VFA, suggesting potential synergistic effects in reducing the risks of type 2 diabetes and visceral obesity. Several mechanisms may explain the beneficial effects of this dietary pattern on glycaemic control and fat accumulation. Pasta, due to its compact microstructure, slows starch hydrolysis (41), leading to reduced glycaemic response and appetite suppression (44). Meanwhile, coffee is rich in polyphenol chlorogenic acid, which has been shown to reduce body fat, particularly visceral fat, possibly by regulating the expression of lipogenic enzymes (45). Although this dietary pattern also includes pizza, Cantonese desserts, and fried foods—many of which are typically associated with increased visceral fat and glucose levels (46–48)—our results suggest that their consumption was generally limited to 1–3 days per week. This occasional intake did not appreciably attenuate the overall benefits of the localised Western dietary pattern in reducing the likelihood of type 2 diabetes and visceral obesity. However, no significant association was found between this dietary pattern and BMI. BMI did not differentiate between fat and lean mass or account for differences in body fat distribution (49). However, visceral obesity, characterised by excessive intra-abdominal adipose tissue accumulation in response to a positive energy balance, is more strongly linked to metabolic abnormalities and is recognised as a major predictor of cardiometabolic disease, independent of BMI and general obesity (50). Specifically, visceral fat accumulation occurs primarily through hypertrophy rather than hyperplasia, which may be attributed to regional differences in preadipocyte replication, differentiation, subtype abundance, susceptibility to apoptosis or senescence, and gene expression (51).

A decreasing trend in hypertriglyceridaemia was observed in relation to modern Cantonese dietary patterns, which was statistically significant in individuals without a prior diagnosis of diabetes. The modern Cantonese dietary pattern includes white meat, eggs, legumes, aquatic products, fresh fruits, vegetables, and milk, which are rich in dietary fibre, protein, and omega-3 polyunsaturated fatty acids (n-3 PUFA). Our findings suggest that this dietary pattern may play a role in reducing visceral fat accumulation. Possible mechanisms include the inhibitory effects of n-3 PUFA on lipogenesis in intra-abdominal fat depots by suppressing key regulators of adipocyte differentiation, such as CCAAT/enhancer-binding protein α and PPARα, thereby limiting adipocyte hypertrophy (52). In addition, dietary fibres may help suppress visceral fat accumulation by promoting the production of short-chain fatty acids (SCFAs) through fibre-degrading gut bacteria (53, 54). SCFAs stimulate the release of the anorectic gut hormones, including peptide YY and glucagon-like peptide-1, which enhance satiety and reduce energy intake (54). However, the synergistic effects of these dietary components on visceral fat accumulation are not yet fully understood. The observed lower likelihood of hypertriglyceridaemia suggests that the modern Cantonese dietary pattern may help reduce the risk of dyslipidaemia. This may be explained by the nutrient composition of this dietary pattern. Dietary proteins are known to lower lipogenic enzyme activity and positively influence lipid metabolism (55). Furthermore, n-3 PUFA, abundant in fresh vegetables and aquatic products, can regulate lipoprotein lipase activity, thereby improving dyslipidaemia (56). In addition, the modern Cantonese dietary pattern occasionally includes Cantonese slow-cooked soup, a traditional dish in Guangdong. Certain herbs commonly used in these soups, such as *Poria cocos*, have been shown to regulate cholesterol homeostasis in hepatocytes via the PPARα pathway, potentially improving hyperlipidaemia and lipid accumulation (57). Moreover, compounds derived from Pinelliae Rhizoma have demonstrated anti-obesity effects (58).

The traditional Cantonese dietary pattern, characterised by the consumption of traditional Chinese ultra-processed foods, appears to be an unhealthy dietary pattern concerning metabolic indicators such as blood lipid levels, FPG levels, and obesity status when compared to the other two dietary patterns. High-quality cohort studies have also demonstrated dose-response relationships between ultra-processed dietary patterns and poor metabolic outcomes (59, 60). The meats consumed in this dietary pattern, including smoked and dried meats, spiced corned products, and roasted meats, are prepared using traditional Chinese processing techniques such as curing, roasting, and brining. This method classifies them as ultra-processed foods (61). Ultra-processed foods not only lose much of their original nutritional quality but also tend to be high in saturated fats, low in fibre and vitamins, and susceptible to the formation of potentially harmful chemicals during processing. These factors may contribute to metabolic disorders and impair tissue and cellular function, which are potentially detrimental to health (62). In our study, Spearman’s correlation analysis revealed that increased intake of ultra-processed foods was associated with elevated LDL-C levels and obesity indicators. These findings underscore the need to monitor ultra-processed food consumption as a strategy to prevent metabolic diseases in Guangdong. In addition to ultra-processed foods, the traditional Cantonese dietary pattern is also characterised by high tea consumption, with 48.2% of participants drinking tea daily. Tea and its bioactive constituents—such as polyphenols, theaflavins, and caffeine—have been shown to potentially inhibit adipose tissue formation, reduce fat mass, improve the insulin/glucose ratio, and exhibit insulin-mimetic activity (63). However, our findings suggest that habitual tea consumption does not attenuate the adverse metabolic effects associated with this dietary pattern.

Our study might have some limitations. First, the cross-sectional design prevents us from inferring causality. However, as individual dietary habits are generally established over long periods, the robustness of our findings was confirmed through sensitivity analysis, which excluded individuals with a prior history of metabolic conditions. Second, although this was a small-to-moderate scale study, we ensured that the sample size met statistical power requirements based on the 10-events-per-variable (10-EPV) principle for sample size estimation (64–66). Third, the data were obtained from the purposive sampling rather than from the Census of Guangdong population; therefore, the interpretation and extrapolation of the study findings should be approached with cautions, as they may be most relevant to individuals who are middle-aged, have resided in Guangdong Province for more than 6 months, and are currently living in Guangzhou. Fourth, the diets were assessed using a self-reported FFQ. Although this method is widely used in epidemiological studies, it might be subject to measurement imprecision and recall bias. Fifth, the study, being a secondary analysis from a published survey, might have omitted certain confounding factors. Future studies with larger populations, more precise dietary intake measurements and long-term follow-ups are needed, to confirm causal relationships and further explore the mechanisms underlying these associations.

## 5 Conclusion

This study characterised the dietary patterns of the South Chinese population, identifying that modern Cantonese dietary patterns appeared to be associated with lower odds of dyslipidaemia, while localised Western dietary patterns might be linked to a reduced likelihood of glucose metabolism disorders or visceral obesity. In contrast, the traditional Cantonese dietary pattern was found to be associated with less favourable metabolic health indicators. These findings provide implications for developing future dietary interventions and highlight the importance of overall dietary patterns in managing metabolic health. However, further studies are needed to establish causality and elucidate the underlying mechanisms to develop effective dietary strategies for preventing metabolic disorders.

## Data Availability

The raw data supporting the conclusions of this article will be made available by the authors, without undue reservation.
